# Internalization and trafficking of CSPG-bound recombinant VAR2CSA lectins in cancer cells

**DOI:** 10.1038/s41598-022-07025-6

**Published:** 2022-02-23

**Authors:** Chris Kedong Wang, Irina Nelepcu, Desmond Hui, Htoo Zarni Oo, Sarah Truong, Sarah Zhao, Zakir Tahiry, Shaghayegh Esfandnia, Fariba Ghaidi, Hans Adomat, Robert Dagil, Tobias Gustavsson, Swati Choudhary, Ali Salanti, Poul H. Sorensen, Nader Al Nakouzi, Mads Daugaard

**Affiliations:** 1 Department of Urologic Sciences, University of British Columbia, Vancouver, BC Canada; 2 Vancouver Prostate Centre, Vancouver, BC Canada; 3 Centre for Medical Parasitology at Department for Immunology and Microbiology, Faculty of Health and Medical Sciences, University of Copenhagen and Department of Infectious Disease, Copenhagen University Hospital, Copenhagen, Denmark; 4 VAR2 Pharmaceuticals, Copenhagen, Denmark; 5 Department of Molecular Oncology, British Columbia Cancer Research Centre, Vancouver, BC Canada

**Keywords:** Cancer, Drug delivery, Cell biology, Glycobiology, Protein transport

## Abstract

Proteoglycans are proteins that are modified with glycosaminoglycan chains. Chondroitin sulfate proteoglycans (CSPGs) are currently being exploited as targets for drug-delivery in various cancer indications, however basic knowledge on how CSPGs are internalized in tumor cells is lacking. In this study we took advantage of a recombinant CSPG-binding lectin VAR2CSA (rVAR2) to track internalization and cell fate of CSPGs in tumor cells. We found that rVAR2 is internalized into cancer cells via multiple internalization mechanisms after initial docking on cell surface CSPGs. Regardless of the internalization pathway used, CSPG-bound rVAR2 was trafficked to the early endosomes in an energy-dependent manner but not further transported to the lysosomal compartment. Instead, internalized CSPG-bound rVAR2 proteins were secreted with exosomes to the extracellular environment in a strictly chondroitin sulfate-dependent manner. In summary, our work describes the cell fate of rVAR2 proteins in tumor cells after initial binding to CSPGs, which can be further used to inform development of rVAR2-drug conjugates and other therapeutics targeting CSPGs.

## Introduction

Endocytosis is the process by which cells internalize a variety of molecules from their surrounding environment. It is a tightly regulated process involving many molecular components acting in synchronization for proper function^1^. There are several endocytic pathways where the most well-knowns are mediated by clathrin, caveolae, and micropinocytosis^1^. Although the different endocytic pathways operate with distinct molecular mechanisms, the general process is similar and involves membrane reshaping to surround materials that subsequently buds off the plasma membrane inside the cell, thereby forming an intracellular endocytic vesicle^1^.

Endocytic vesicles are transported inside the cells to different organelle compartments depending on their cargo^2^. The first compartment where almost all vesicles are trafficked to is the early endosome (EE). The EE is a mildly acidic (~ pH6) compartment and contains enzymes that can facilitate the release of ligands from receptors, which can then be recycled back to the cell surface^3^. The EE is the primary organelle where cargo is sorted for subsequent transport to other compartments. Some cargo will be packed into sub-organelle vesicles by the inward budding of the endosomal membrane into the lumen, which is then pinched off to form intraluminal vesicles (ILV)^4^. Endosomes that contain ILVs are known as multivesicular bodies (MVB). MVBs are trafficked to the cell surface where they fuse with the plasma membrane to release their content, including ILVs, to the surroundings^4^. ILVs secreted outside the cell are commonly referred to as exosomes^4^. Cargo that is not enclosed into ILVs will leave the EE and proceed to the late endosome (LE) compartment. The LE is slightly more acidic (~ pH5.5) than the EE and is the last cargo sorting site prior to delivery to lysosomes^5^. Lysosomes are the final compartment for materials destined for degradation. They are very acidic (~ pH4) and contain hydrolases that break down organic molecules to their basic subunits, which are then used by the cell as building materials^6^.

Each endocytic compartment has unique biochemical properties and enzymes that directly influence the release and activity of a given ligand^7^. It is therefore pertinent to understand the intracellular trafficking pathway for a drug delivery vehicle to achieve optimal activity of the drug. Antibody–drug conjugates (ADC) are a group of drugs that exploits the specific antigen binding capabilities of antibodies for targeted drug delivery^8^. As the number of known cancer-specific targets have increased dramatically in the past decade, opportunities for ADC development have followed and several ADCs have been approved for treatment of breast cancer, lymphoma, and other cancer types, with many more in development^9,10^. ADCs are typically comprised of a target-specific antibody attached to a cytotoxic drug through a linker^8^. The antibody serves to target and deliver the drug-conjugate directly to the cancer cells while limiting drug exposure in healthy tissues. When bound and internalized inside the cancer cells, the linker usually determines into which intracellular compartment the drug is released^9,10^. The drug itself is usually designed to be activated under certain conditions only, for example in the acidic environment found in the lysosome. By taking these steps into consideration, drug-conjugates can in theory be specifically designed to have high efficacy with limited toxicity.

In addition to antibodies, other molecules targeting cancer cell biomarkers are under development. We have, for example, developed a recombinant malarial protein, rVAR2, that is capable of binding a unique oncofetal chondroitin sulfate (ofCS) on the cancer cell surface^11–13^. Besides cancer cells, ofCS has only been found on the surface of syncytiotrophoblast in the placenta of pregnant women^14–16^. The ofCS is part of a family of linear unbranching glycosamino glycans (GAGs) that are typically found in the extracellular matrix (ECM) or attached to proteoglycans on the cell surface^17–19^. GAGs are comprised of long chains of disaccharide subunits that typically contains an amino sugar and a uronic acid^20^. There are several different types of each sugar, which give rise to different combinations of disaccharides^20^. GAG chains can be further modified with sulfate groups attached to specific sites on GAG chains^21^. This provides cells with the ability to create diverse GAG compositions that are used under specific cellular circumstances^21^. One of the most common GAGs are chondroitin sulfate (CS)^11^ that is comprised of repeating *N*-acetylgalactosamine (GalNac) and glucuronic acid (GlcA) disaccharide subunits. CS chains can be modified by the addition of sulfate groups on the 4-*O* and 6-*O* positions of the GalNac residue. The degree to which the CS chain is sulfated varies between tissues and pathologies. ofCS is unique in that almost the entire CS chain carries the 4-*O* sulfation. rVAR2 can specifically target and bind ofCS on cancer cells and has also been used in the development of rVAR2 drug conjugates (VDCs)^11,12^. Some of these VDCs were shown to be effective against drug-resistant bladder cancer in vivo model^11,12^. However, the optimal VDC design for maximum anti-tumor efficacy, depends in part on the internalization and trafficking pathways of the rVAR2 protein. In this study, we have investigated the endocytic trafficking pathways of rVAR2 within cancer cells to expand our knowhow on cellular trafficking of GAGs that can potentially improve VDC designs and rVAR2-based therapies.

## Results

### rVAR2 proteins are internalized into cancer cells

**Figure 1:**
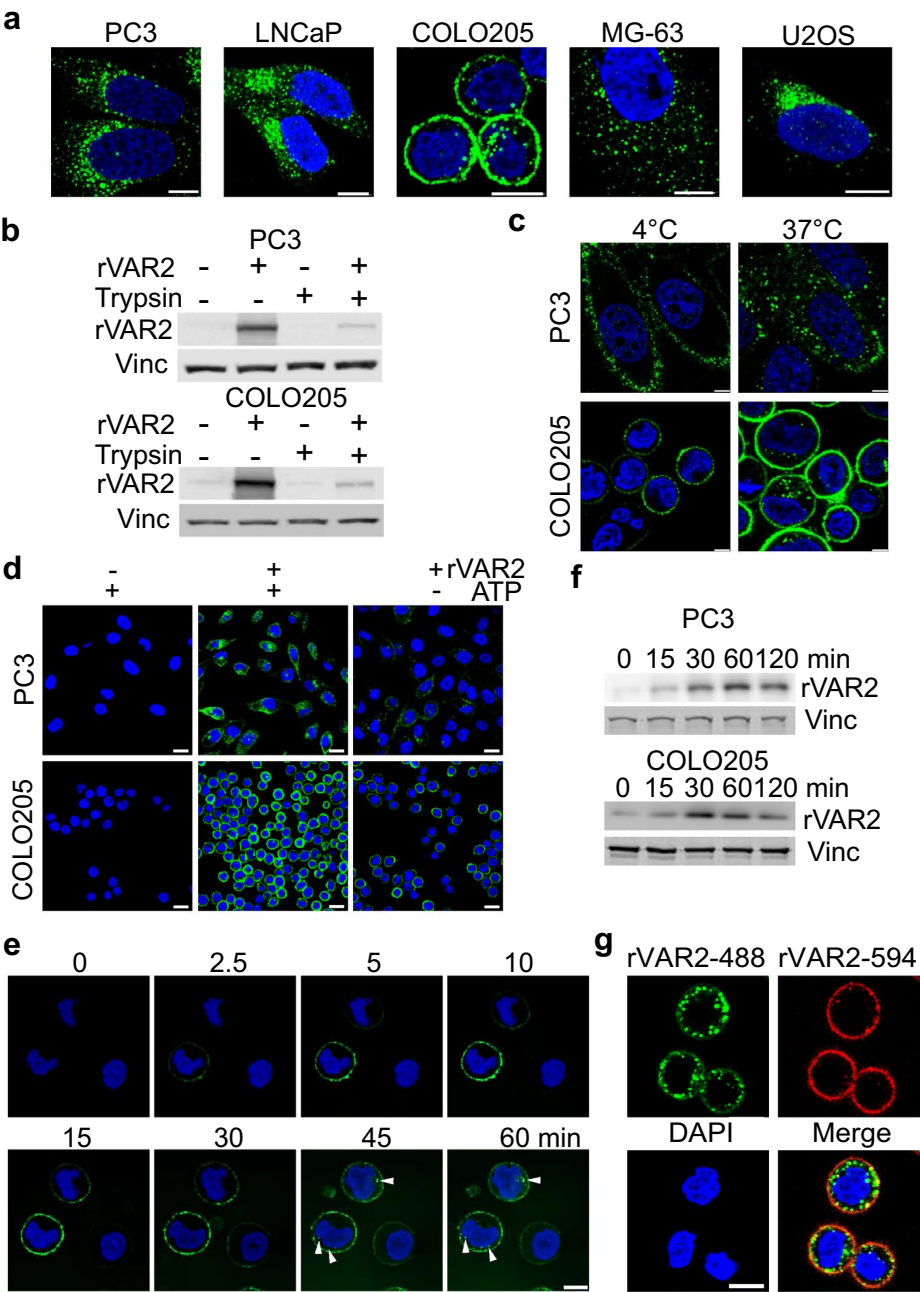
rVAR2 is internalized into cancer cells. (**a**) Confocal images of internalized rVAR2 in cancer cell lines after 2 h of treatment. (**b**) Western blot for rVAR2 in PC3 and COLO205 cells from lysates collected by either direct lysis on the plate or trypsinizing the cells. (**c**) Confocal images of cells treated with rVAR2 at 4 °C or 37 °C. (**d**) Analysis of rVAR2 binding with or without ATP depletion in PC3 and COLO205 cells. (**e**) Representative images from live cell confocal of rVAR2 internalization in COLO205 cells, arrow heads indicate the presence of rVAR2 inside cells. (**f**) Western blot of rVAR2 accumulation in PC3 and COLO205 cells over time. Vinculin was used as loading control. (**g**) COLO205 cells were pulse treated with rVAR2-488 and allowed to be internalized for 2 h prior to rVAR2-594. Scale bar represents 5 µm.

Cell lines representing different cancer indications were treated with rVAR2 and imaged using confocal microscopy. After 2 h of treatment, rVAR2 was observed as clusters inside the cytoplasm of cells originating from prostate cancer (PC3 and LNCaP), osteosarcoma, (MG-63 and U2OS), and colon cancer (COLO205) (Fig. 1a). To confirm rVAR2 internalization into the cytoplasm, PC3 and COLO205 cells were incubated with rVAR2 for 1 h and lysates were collected after extended trypsin treatment to remove plasma membrane CS-modified proteoglycans (CSPG) (Fig. 1b). There was a marked decrease of detectable rVAR2 in trypsinized cells indicating that most rVAR2 was still bound to the trypsin-accessible cell surface or surrounding ECM after 1 h. However, a portion of rVAR2 remained present after trypsin supporting the observation that some rVAR2 protein gets internalized into cancer cells (Fig. 1b). We next investigated whether the rVAR2 internalization was an energy dependent process. Human cancer cell lines PC3 and COLO205 were treated with rVAR2 for 30 min at 4 °C or 37 °C followed by an acid wash to remove surface-bound recombinant rVAR2 before being analyzed by confocal microscopy. Localization of rVAR2 was determined by immunofluorescence staining using a monoclonal antibody against V5-tag. Punctate rVAR2-positive staining (green) was observed at 37 °C, but was not detected at 4 °C (Fig. 1c). Pretreatment of cells with sodium azide and 2-deoxy-d-glucose to deplete the intracellular ATP pool caused a significant decrease in rVAR2 uptake (Fig. 1d). These results suggest that endocytosis of rVAR2 is both temperature sensitive and energy dependent. Next, we investigated the kinetic profile of rVAR2 internalization for up to 2 h after rVAR2 treatment. Using live cell real-time confocal microscopy, we observed rVAR2 binding to COLO205 cells within 10 min and internalization starting 30 min after treatment (Fig. 1e). This observation was confirmed by immunoblotting for rVAR2 in PC3 and COLO205 cells where rVAR2 accumulated over time in lysate from trypsinized cells (Fig. 1f). We then tested whether rVAR2 gets internalized continuously after binding to cells by using two fluorophores conjugated to rVAR2 (rVAR2-488 and rVAR2-594). We first pulse-treated COLO205 cells with rVAR2-488 for 15 min then replaced the media with normal media for 2 h to allow all bound rVAR2 to be internalized. We then followed this up immediately with a 15 min rVAR2-594 pulse treatment. At this time point, rVAR2-488 was present within the cytoplasm with trace amounts still bound to the plasma membrane. Importantly, while rVAR2-594 was largely bound to the membrane (Fig. 1g), it co-localized with rVAR2-488 in intracellular structures near the cell surface (Fig. 1g). Combined, these data show that rVAR2 gets internalized into cancer cells in a time and energy-dependent manner.

### Cell surface CS expression is required for rVAR2 uptake in cancer cells

**Figure 2:**
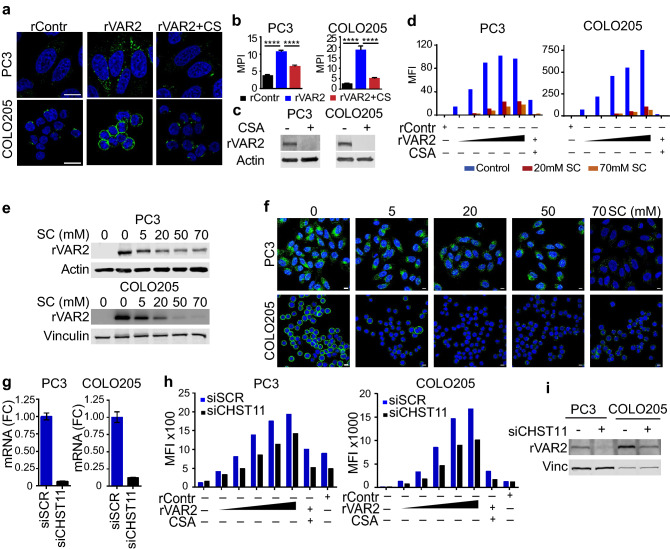
rVAR2 internalization depends on binding to cell surface CSA. (**a**) Immunofluorescence of rVAR2 in COLO205 and PC3 cells after 2 h incubation. **(b)** quantification for mean pixel intensity of AlexFluor488 signals in rContr, rVAR2, and rVAR2+ CS treated cells. Statistical analysis was performed on n ≥ 30 cells, ****p ≤ 0.0001 by ANOVA followed by Tukey’s post hoc analysis. Scale bar represents 10 µm. (**c**) Western blot for intracellular rVAR2 follow rVAR2 treatment in the presence or absence of CSA competition. (**d**) Flow cytometry analysis of rVAR2 binding in COLO205 and PC3 cells with and without SC treatment. (**e**) Western blot of internalized rVAR2 in COLO205 and PC3 cells after SC treatment at the indicated concentrations. rVAR2 was incubated for 1 h and vinculin was used as loading control. (**f**) Immunofluorescence images of rVAR2 after 1 h incubation in COLO205 and PC3 cells following SC treatment. Scale bar represents 10 µM. (**g**) CHST11 mRNA levels in PC3 and COLO205 cells as determined by qPCR and normalized to GAPDH. (**h**) rVAR2 binding analysis 48 h following control and CHST11 siRNA transfections in PC3 and COLO205 cells. (**i**) Western blot of internalized rVAR2 in PC3 and COLO205 cells following CHST11 knockdown, vinculin was used as loading control.

Since rVAR2 can potentially enter the cell through non-specific endocytosis, we next examined whether rVAR2 internalization is dependent on cell surface CS binding. First, we introduced purified CSA into the media to compete for rVAR2 binding. Purified CSA is comprised of different populations of mainly carbon-4 sulfated (C4S) CS that determines rVAR2 specificity^11^. Cellular rVAR2 binding and internalization in COLO205 and PC3 cells were effectively out-competed by addition of purified CSA as assessed by confocal microscopy (Fig. 2a,b) and immunoblotting (Fig. 2c). The data suggest that the binding and internalization requires interaction between rVAR2 and CS. To investigate whether rVAR2 binding to the cells depends on sulfation, an rVAR2-binding assay was carried out in the presence of sodium chlorate (SC). SC competitively inhibits the formation of 3′-phosphoadenosine 5′-phosphosulfate^22,23^, which is the high energy sulfate donor in cellular sulfation reactions thereby preventing sulfation of glycoproteins and carbohydrates. PC3 and COLO205 cells grown in medium containing SC for 24 h had reduced binding to rVAR2 as determined by flow cytometry (Fig. 2d). Internalized rVAR2 was markedly reduced in SC treated cells analyzed by immunoblotting (Fig. 1e), and confirmed by immunofluorescence (Fig. 2f). Furthermore, downregulation of the C4S sulfotransferase CHST11 (Fig. 2g) decreased rVAR2 binding assessed by flow cytometry (Fig. 2h) and internalization assessed by immunoblotting (Fig. 2i) in both PC3 and COLO205 cell lines. Downregulation of CHST11 did not alter the expression of other C4S (CHST12/13) and C6S (CHST3/7) chondroitin sulfotransferases (Fig. S2a). In addition, CHST11 appears to be the most highly expressed C4S chondroitin sulfotransferase in both PC3 and COLO205 cells (Fig. S2b). Linear regression analysis of rVAR2 binding after CHST11 downregulation showed a significant reduction in rVAR2 binding (Fig S2b). In summary, these data indicate that cell surface CS is crucial for the interaction with rVAR2 and its subsequent internalization.

### Internalization of rVAR2 is cell energy-dependent and can occur via different endocytic pathways

**Figure 3:**
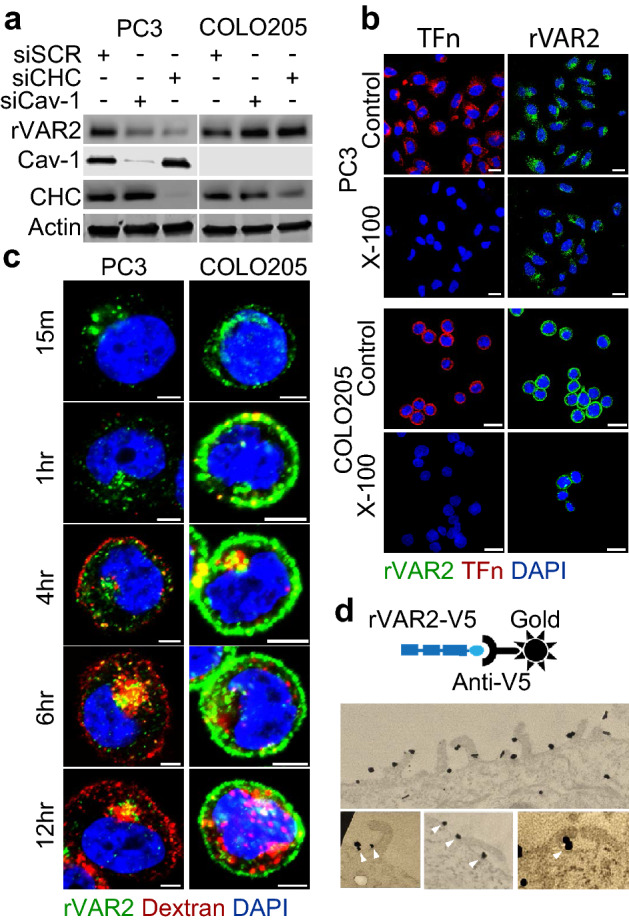
Endocytic mechanism of rVAR2. (**a**) Western blot for rVAR2, CHC, and Cav-1 in PC3 and COLO205 cells following transfection with siSCR, siCHC, or siCav-1. Actin was used as loading control. (**b**) Confocal images of rVAR2 in Triton X-100 treated PC3 and COLO205 cells, transferrin (TFn) was used as control for Triton X-100 treatment. Scale bar represents 10 µM. (**c**) Colocalization of rVAR-488 and Dextran-594 in COLO205 and PC3 cells at indicated timepoints. Scale bar represents 5 µM. (**d**) Electron microscopy of COLO205 cells treated with rVAR2. Gold-nanoparticle labeled antibodies was used to detect the presence of rVAR2. Arrowheads indicate the presence of rVAR2 binding near membrane protrusions that corresponds to structures formed during macropinocytosis.

Endocytic pathways can be classified into three classes: (i) endocytic pathways that take place in lipid rafts, which include the majority of clathrin-independent endocytic pathways such as caveolae-mediated endocytosis^24,25^; (ii) pathways that do not involve lipid rafts in the endocytic vesicle, namely clathrin-mediated endocytosis (CME)^2,24^; and (iii) endocytic pathways for which the endocytic vesicle can contain lipid rafts together with non-raft membrane domains, and these include phagocytosis and micropinocytosis^24,26^. Amongst the many endocytic mechanism cells can use to internalize external materials, two major routes are clathrin-mediated and caveolae-mediated endocytosis (CavME)^1^. They are both receptor dependent and require coat proteins originating from the cell membrane to form vesicles. Because of this, clathrin and caveolae mediated endocytosis typically occurs when specific triggers are present^2,27–29^. First, we investigated whether rVAR2 is internalized through clathrin and caveolae mediated pathway by using siRNAs to downregulate clathrin (CHC) and caveolin-1 (Cav-1) coat proteins in PC3 and COLO205 cells (Fig. 3a). In PC3 cells, knockdown of CHC and Cav-1 decreased rVAR2 accumulation, suggesting that rVAR2 can be internalized through other pathways. While in COLO205 cells, the absence of Cav-1 expression or CHC downregulation did not significantly alter rVAR2 internalization. These observations would suggests that CME and CavME are not key players for rVAR2 internalization in COLO205 cells. Clathrin-mediated endocytosis pathways are lipid rafts-independent and lipid rafts are resistant to detergent solubilization at low temperatures^30,31^. To further correlate the association of rVAR2 with clathirin, rVAR2-treated PC3 and COLO205 cells were subsequently treated with ice-cold 1% (v/v) Triton-X-100 for 3 min to extract non-raft-localized proteins^30^. After fixation, cells were examined by confocal microscopy using the lipid-raft marker transferrin (TFn) as a control. In both cell lines, TFn was only detected in the control setting while the rVAR2 signal remained after Triton-X-100 extraction, suggesting that internalization of rVAR2 is also independent of clathrin (Fig. 3b).

Since internalization of rVAR2 appears to be not entirely independent of both clathrin- and caveolae-mediated pathways, we next investigated whether internalization of rVAR2 could be associated with macropinocytosis (MP). Macropinocytosis is a more general internalization pathway driven by reorganization of actin filaments in the cytoplasm that form membrane protrusions ascending from the plasma membrane that folds back onto itself to create vesicles^26,32^. The formation of vesicles through macropinocytosis is a well-defined process that forms easily recognizable structures on the cell plasma membranes^33,34^. Unlike receptor mediated endocytosis, macropinocytosis is not triggered by specific ligand-receptor interactions, but is rather triggered by general binding of materials to the cell surface. Since rVAR2 binds CS chains rather than specific protein receptors, it is possible that rVAR2 might be internalized though macropinocytosis. First, we tested whether rVAR2 co-localizes with dextran, a known marker for micropinocytosis^33–36^. COLO205 and PC3 cells were treated simultaneously with AlexaFluor 594-labeled dextran (Dextran-594) and rVAR2-488 and harvested at different timepoints. Indeed, rVAR2 and dextran co-localized inside cells, indicating they are in the same compartment (Fig. 3c). Moreover, rVAR2 associated with unique macropinocytosis structures on the cell membrane, as assessed by transmission electron microscopy (Fig. 3d). Combined, these data suggest that rVAR2 can utilize several endocytic mechanisms to enter the cell and that the choice of endocytosis pathway is cell line dependent.

### Internalized rVAR2 is trafficked to early-endosomes but not lysosomes

**Figure 4:**
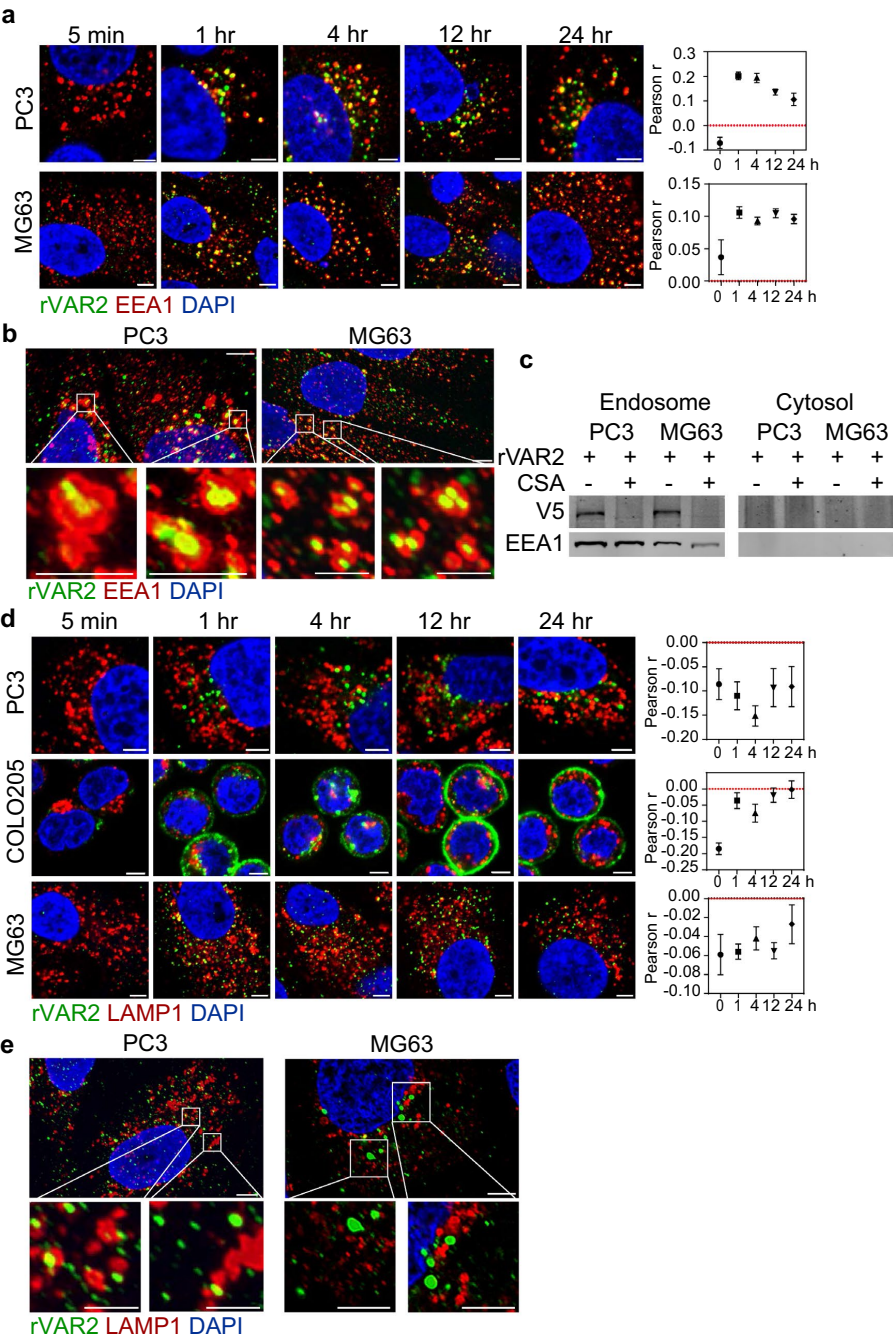
Intracellular trafficking of rVAR2. Confocal images of PC3, COLO205, and MG63 cells stained for rVAR2 and (**a**) EEA1 or (**d**) LAMP1 after rVAR2 incubation at the indicated timepoints. Colocalization was quantified by measuring pearson correlation coefficient of AF488 (rVAR2) and AF594 (markers) signals from confocal images. Values represents the average ± standard error of ≥ 10 images. High resolution images of rVAR2 colocalizing with (**b**) EEA1 or (**e**) LAMP1 were acquired in cells after 4 h of rVAR2 incubation. Scale bar represents 5 µm in large images and 500 nm in small images. **(c)** Western blot of purified endosome or cytosol by subcellular fractionation in PC3 and MG63 cells.

We next investigated the rVAR2 cell fate following internalization. Different cellular compartments have distinct biochemical properties (e.g., pH, enzyme compositions, or chemical content) that potentially affect protein stability, as well as VDC drug release and efficacy. To determine the cell fate of VAR2 after internalization, tumor cells (PC3, COLO205, and MG63) were treated with rVAR2 for up to 24 h at 37 °C and examined by confocal microscopy for rVAR2 co-localization with markers of the early endosome (EEA1) and lysosome (LAMP1). However, COLO205 cells were reported to carry a frameshift mutation for EEA1 therefore we cannot characterize rVAR2 and EEA1 colocalization in this cell line^37^. Instead, we decided to introduce MG63 as a second cell line to verify EEA1 colocalization. rVAR2 co-localized with EEA1 in PC3 and MG63 cells within 1 h and up to 24 h of continuous treatment (Fig. 4a, *left*). The analysis showed a positive correlation between rVAR2 and EEA1 (Fig. 4a, *right*). High resolution imagining confirmed the presence of rVAR2 in the lumen of the EEs (Fig. 4b). In addition, isolated endosomes fractions from rVAR2-treated PC3 and MG63, contained rVAR2 protein, as assessed by immunoblotting (Fig. 4c). Contrarily, rVAR2 was not found co-localizing with the lysosomal marker LAMP1 at any time point in any of the three cell lines (Fig. 4d,e). Taken together, these results suggest that rVAR2 is trafficked to the EE after internalization but is not passed on to the lysosome.

### Internalized rVAR2 is ultimately released outside cells with exosomes

CSPGs are a diverse family of proteins some of which can act as cell surface receptors. Cell surface receptors and other integral membrane proteins are internalized by endocytosis and routed to early-endosomes. From there, the proteins are recycled back to the plasma membrane, or are sorted into intraluminal vesicles (ILV) that form through inward budding of endosomes and give rise to multivesicular bodies (MVB). Fusion of MVBs with lysosomes results in degradation of ILVs and their cargo, whereas fusion of MVBs with the plasma membrane results in release of ILVs as exosomes^4^.

**Figure 5:**
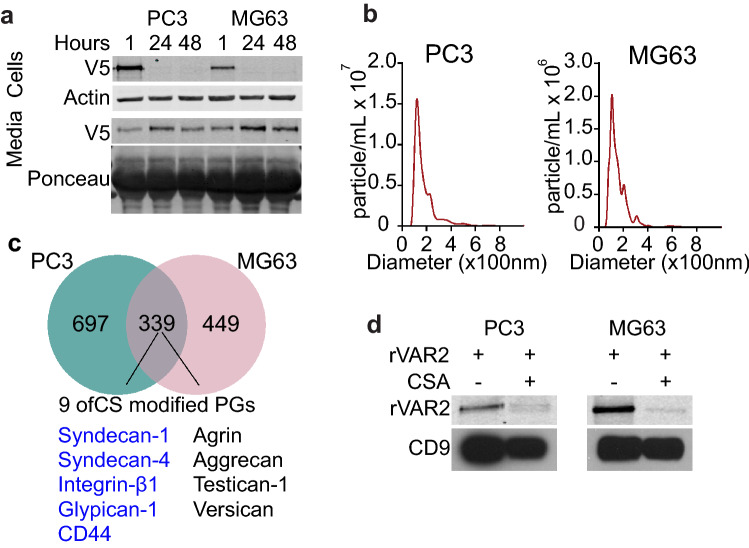
rVAR2 is released outside cells with exosomes. **(a)** PC3 and MG63 were treated with rVAR2, trypsinized lysated or media was collected at the indicated timepoints and blotted for the presence of rVAR2. Vinculin was used as loading control for cell samples and ponceau red stain was used as loading control for media samples. **(b)** Nanoparticle tracking analysis (NTA) of exosomes purified from the media of PC3 or MG63 cells. **(c)** Proteome of PC3 or MG63 exosomes as determined by mass spectrometry, common proteins present in exosomes from both cell lines are highlighted. CSPGs found in the exosomes of both cell lines are listed and known cell surface CSPGs are listed in blue. **(d)** Western blot of exosomes from PC3 or MG63 cells 48 h after rVAR2 treatment. 20 billion particles were used for loading and CD9 was used to determine the presence of exosomes.

As internalized rVAR2 does not reach the lysosome (Fig. 4d,e) and appears to decrease after 2 h (Fig. 1f), it is possible that rVAR2 might be secreted from the cells. To investigate if rVAR2 is secreted outside the cells, PC3 and MG63 cells were treated with rVAR2 for 1 h, washed with PBS thrice and re-supplied complete media. Cells and media were collected at 1, 24 and 48 h and analyzed by immunoblotting for rVAR2 presence. We observed that rVAR2 was present in the cell lysate at 1 h but disappeared after 24 h (Fig. 5a). This coincided with an increase of rVAR2 in the media after 24 h and 48 h in both cell lines, supporting the idea that rVAR2 might be shuttled outside the cells after reaching the endosome.

Next, we tested whether internalized rVAR2 is subsequently secreted with exosomes. We purified exosomes from both PC3 and MG63 cells and confirmed the exosome fraction by determining the nanoparticle size-distribution using Nanoparticle Tracking Analysis (NTA, NanoSight). The samples contained > 90% particles with a size of 50–150 nm (Fig. 5b), consistent with the size of exosomes. We analyzed the protein content of the exosome fractions by mass spectrometry. Among the 330 common proteins identified in the exosome fractions of the two cell lines, 9 of them were proteoglycans which some have previously been reported to express ofCS and 5 of these proteoglycans are expressed on the cell surface (Fig. 5c)^38^. To assess the presence of rVAR2 in the exosome fraction, both cell lines were treated with rVAR2 with and without CSA competition for 1 h. The cell culture plates were washed thrice with PBS to remove unbound rVAR2 and fresh exosome depleted media were added. Cell culture media was subsequently collected at 24 h for exosome extraction. Immunoblotting analysis of exosome lysates showed that rVAR2 was present in the exosomes fraction of both cell lines (Fig. 5d). Importantly, the presence of rVAR2 in the exosome fraction was inhibited by CSA competition, indicating that rVAR2 needs to be internalized by the cells in order to appear with exosomes. In summary, these data suggest that internalized rVAR2 is secreted from the cells in the exosome fraction.

## Discussion

In the present study we investigated the internalization process of the non-endogenous CS-binding lectin rVAR2, currently in development by the industry as a drug-delivery system for treatment of multiple cancer indications. rVAR2 is reported to bind a type of CSA with oncofetal specificity to placental- and cancer cells. Much like an ADC, rVAR2 can be formulated as a VDC with different payloads to target ofCS-positive tumors^11,12^. Most solid tumors express medium to high levels of ofCS and a first generation VDC has shown promise of reducing tumor burden in several in vivo models^11,12^. However, to understand and further improve VDC efficacy, understanding how rVAR2 proteins are trafficked in cancer cells is important.

Our data confirmed cell surface CS as the true endocytic receptors of rVAR2 rather than co-receptors, which only promote attachment. We demonstrated that various endocytic mechanisms, such as CavME, CME, and MP are involved in rVAR2 internalization into tumor cells. Due to the functional diversity of CSPGs, it is not surprising that several endocytic pathways contribute to the internalization. Which rVAR2 endocytic mechanism that is most active in individual cells likely dependent on CSPG context. Our data suggest that regardless of the endocytic pathway used, rVAR2 ultimately reach the EE without further trafficking to lysosomes in PC3, MG63 and COLO205 cells. When quantified, we observed that colocalization of rVAR2 and EEA1 decreased linearly over time while remaining constant in MG63 cells. This is an interesting observation which might be due to the differences in CS expression or recycling between the two cell lines. The characteristics of the ligand which binds to CS could be a significant factor in determining the endocytosis route and intracellular trafficking itinerary. It remains to be determined how the endocytic pathway and the fate of rVAR2 depend on its own characteristics. The common trafficking fate of rVAR2 would impact the design of next generations of VDCs. Linkers and toxins that have the maximum efficacy in the conditions of the early endosome would be the logical choice. For example, Furin is a protease that is found in the early endosome and studies have shown that ADCs with a furin-cleavable linker in drug conjugates can enhance anti-tumor efficacy^39,40^. Incorporating the findings from this study could potentially lead to more effective VDC designs that maximize anti-tumor efficacy tailored to each malignancy^39,40^.

While rVAR2 is not trafficked to the lysosome, our data indicate that it is secreted outside the cell with exosomes. It is possible that rVAR2 could be associated with the surface of the exosomes since they are formed by the inward budding of the endosomal membrane. The receptor for rVAR2 is ofCS on CSPGs and our data show that cell membrane associated CSPGs are indeed present in exosome fractions from tumor cells. Therefore, rVAR2 might remain bound to ofCS on exosomes and simply follow the same trafficking/secretion pathway as exosomes. Another possibility is that after rVAR2 reaches the early endosome, it is shed from the CSPGs through enzymes that degrade CS. A similar mechanism for heparan sulfate shedding for exosome formation has been suggested^41^.

Previous studies have shown that exosomes may function as cellular waste disposal that expel nonfunctional/foreign components^42,43^. Being a non-human lectin, it is possible that rVAR2 is expelled from the cells in a similar manner. A wide range of functions for exosomes in biology and tumorigenesis has been proposed. Supporting that idea, we found that cancer-derived exosomes from two different cell lines express common CSPGs. It is tempting to speculate that there might be a common function for these CSPGs in the context of exosomes. Further studies on the role of CSPGs in the context of cancer-derived exosomes are needed to illuminate their roles in cancer biology. Indeed, exosomes has been shown to be involved in metastasis and in preparation of the pre-metastatic niche^44,45^. Given the role of cell surface CSPGs in metastasis^46–49^, it is possible that exosomal CSPGs contribute to the described exosome-driven metastatic phenotype.

## Methods

### Cell line and culturing condition

PC3, COLO205, LNCaP, U2OS, and MG63 cells were procured from ATCC (Manassas, VA, USA). PC3 and U2OS cells were maintained in DMEM, COLO205 was maintained in RPMI, and MG63 maintained in MEM media. All media were supplemented with 10% FBS and grown in 5% CO2 at 37 °C. All cells were tested for mycoplasma using the MycoAlert Mycoplasma detection kit from Lonza Bioscience (cat# LT07-118).

### Quantitative PCR

RNA extraction was done using TRIzol^®^ RNA Isolation Reagents (Invitrogen Life Technologies, Inc). 2 μg of RNA was reversed transcribed using MMLV reverse transcriptase and random hexamers (Invitrogen) according to manufacture instructions. PCR primers and probes for the following genes were purchased from Life Technology and used according to the manufacturer’s recommendations. qPCR was performed on an ABI ViiA™ 7 Real‐Time PCR detection system (Applied Biosystems) with a SyberGreen ROX master mix (Roche). The amount of sample RNA was normalized by the amplification of glyceraldehyde‐3‐phosphate dehydrogenase (GAPDH) levels as internal control. The results are representative of at least three independent experiments. qPCR primer sequences for genes are: CHST11 *forward: TTTCCAAATCATGCGGAGG reverse: AGGACAGCAGTGTTTGAGAG*, GAPDH *forward: ACCCAGAAGACTGTGGATGG reverse: CAGTGACTTCCCGTTCAG*.

### Western blot

Cells were grown to 80% confluency then treated with 50 nM of rVAR2. Whole‐cell lysates were obtained by lysing the cells in an appropriate volume of ice‐cold RIPA buffer composed of 50 mmol/l Tris–HCl, pH 7.4, 150 mmol/l NaCl, 0.5% sodium deoxycholate, 1% Nonidet P‐40, 0.1% SDS containing 1 mmol/l Na3VO4, 1 mmol/l NaF, 1 mmol/l phenylmethylsulfonylfluoride, and protease inhibitor cocktail tablets (Roche Applied Science). Cellular extracts were clarified by centrifugation at 13,000×*g* for 10 min and protein concentrations was determined by a BCA protein assay kit (ThermoFisher). Thirty micrograms of the extracts ware boiled for 5 min in SDS reducing sample buffer, separated by SDS–PAGE, and transferred onto a nitrocellulose membrane following standard methods. Membranes were probed with dilutions of primary antibodies CHC (Cat# 2410S) and Cav-1 (Cat# 3238S) from Cell Signaling, V5 (Cat# R96025) from ThermoFisher, and Actin (Cat# A2228-100UL) and vinculin (Cat# V4505) from Sigma, followed by incubation with either horseradish peroxidase‐conjugated secondary antibodies or fluorescent secondary antibodies. After washing, proteins were visualized by either a chemiluminescent detection system (GE Healthcare) or Odyssey Imaging System (LI‐COR).

For exosome western blots, 20 billion particles were concentrated using Amicon Ultra-0.5 centrifugal filter unit (Sigma Cat# UFC501024) according to manufacturer instructions. Concentrated exosomes were lysed using reducing sample buffer (0.25 M Tris HCl (pH 6.8), 40% glycerol, 8% SDS, 5% 2-mercaptoethanol and 0.04% bromophenol blue) and boiled for 10 min at 95 °C. Proteins were resolved as previously described. Membrane were probed with antibodies against CD9 (Abcam Cat# ab223052) and V5 (ThermoFisher Cat# R96025).

### RNA interference

siRNAs in this study were purchased from Qiagen and transfected in to cells using RNAiMAX (ThermoFisher Cat# 13778075) according to manufacture instructions. 20 nM of siRNA was used for knockdown. Subsequent experiments were performed 48 h post transfection to endure adequate knockdown of protein expression. The siRNA used were: Scramble (Cat# 1027310), CHC (SI02651747), Cav-1 (SI02731813), and CHST11 (SI04290958).

### Flow cytometry binding assay

PC3 and COLO205 cells were grown to 80% confluency in the appropriate growth media and harvested in an EDTA detachment solution (Cellstripper, Corning Cat# MT25056CI). Cells were incubated with rVAR2 (12.5–200 nM) in PBS containing 2% fetal bovine serum (FBS) for 1 h on ice and binding was analyzed in a FACSCantoII (BD Biosciences) after a 1 h secondary incubation with an anti-V5-FITC antibody (Invitrogen Cat# R963-25). For CSA competition, cells were co-incubated with rVAR2 and 400 μg/ml purified CSA (Sigma Cat# C6737-5G). The data was analyzed using FlowJo V10.4.2.

### Confocal imaging

Cells were grown to 60% confluency on glass coverslips, then incubated with rVAR2 at the indicated time points (5 min to 24 h) prior to processing. Cells were fixed in 4% paraformaldehyde (PFA) for 15 min at RT, permeabilized with 0.05% triton-X100 for 5 min and blocked in 3%BSA for 30 min. Primary antibodies against EEA1 (Cell signaling Cat# 3288S), LAMP1 (Cell Signaling Cat# 9091S), and V5 (ThermoFisher Cat# R96025) were diluted at 1:100 in blocking buffer and incubated overnight at 4 °C in a dark humidifier chamber. The cells were washed thrice in PBS and incubated with secondary antibodies diluted at 1:250 in blocking buffer and 1 h at RT in the dark. The coverslip was washed thrice with PBS then mounted to cover slide with VECTASHIELD mounting media with DAPI (Vector Lab. H-1200-10). Images were taken on a Zeiss LSM 780 confocal microscope at × 63 magnification.

### Live cell confocal

COLO205 cells were grown to 80% confluency in the appropriate growth media on glass bottom dishes (MatTek Cat# P35G-1.5-14-C). The Nucleus was stained with 1:1000 dilution of Hoechst 33342 (ThermoFisher Cat# H3570) 15 min prior to incubation with 20 nM of rVAR2 directly labeled with an AlexaFluor-488 dye (rVAR2-488). Images were taken at 30 s intervals on a Zeiss Cell Observer spinning disc confocal microscope (Zeiss) using a 63× oil immersion lens. Still images were exported at the indicated time points.

### High resolution confocal

Cells were grown to 60% confluency on clover slides, then incubated with rVAR2 at the indicated time points and Hoechst 33342 15 min prior to fixation. Cells were fixed in 4% paraformaldehyde (PFA) for 15 min at RT, permeabilized and blocked in 3% BSA/0.1% saponin for 30 min. Primary antibodies against EEA1 (Cell signaling Cat# 3288S), LAMP1 (Cell Signaling Cat# 9091S), and V5 (ThermoFisher Cat# R96025) were diluted at 1:100 in blocking buffer and incubated overnight at 4 °C in a dark humidifier chamber. Secondary antibodies were diluted at 1:250 in the blocking buffer and incubated on cells for 1 h at RT in the dark. The cover slips were washed then mounted to cover slide with Prolong glass antifade mounting solution (ThermoFisher Cat# P36982) and left to cure for 24 h. Z-stack Images were captured on an Olympus FLUOVIEW FV3000 confocal with high-sensitivity detectors followed by advanced constrained iterative deconvolution.

### Colocalization analysis

Colocalization was measured using the co-localization module in Zen 2013 (Black edition) software. An entire field of view is analyzed on a pixel-by-pixel basis. A modified pearson’s correlation coefficient (R) is generated to determine the colocalization of two channels. R = 1 represents perfect correlation where a pixel with 488 will always contain 594. R is reported for an entire image of 1024 px by 1024 px.

### Electron microscopy

COLO205 cells were grown on aclar disks treated with rVAR2 for 1 h prior to fixation with solution of 4% EM-grade PFA in 0.1 M sodium cacodylate for 1 h at RT. Cells were permeabilized and blocked in 3% BSA/0.1% saponin for 30 min. Primary antibody against V5 (ThermoFisher Cat# R96025) were diluted 1:50 in blocking buffer and incubated for 1 h RT in a dark humidifier chamber. Secondary antibody with gold nano particles (Nanoprobes Cat# 7001—0.5 ml) were diluted in the blocking buffer at 1:100 and incubated on cells for 1 h at RT in a dark humidifier chamber. Cells were then processed with 1% osmium tetroxide for 1 h. Following this, cells were dehydrated using graded ethanol, and were then infiltrated, embedded, and polymerized in eponate resin. Ultrathin sections were cut and stained with 5% uranyl acetate and 2% lead citrate. Images were acquired on a Hitachi H7600.

### Endosome isolation

PC3 and MG63 cells were grown to 80% confluency then treated with rVAR2 for 1 h at 50 nM. Endosome and cytoplasmic fractions were collected using Minute™ Endosome Isolation and Cell Fractionation Kit (Invent Biotechnologies Cat# ED-028) according to manufacturer instructions. Extracts were analyzed using western blotting.

### Exosome isolation and characterization

PC3 and MG63 cells were procured grown in normal media at at 37 °C and 5% CO2. At 50% confluency in 150 mm culture dishes, cells were washed thrice with PBS then supplemented with media containing 5% exosomes depleted FBS (System Biosciences Cat# EXO-FBS-250A-1). After 48 h, culture supernatant was collected and centrifuged twice at 1000×*g* for 10 min to remove cellular debris. 15 ml of cleared media was then concentrated to ~ 1 ml by centrifugation at 14,000×*g* for 30 min using Amicon^®^ Ultra-15 centrifugal filter unit (Sigma Cat# UFC901008). Concentrated media was then was overlaid on 35 nm qEV-original size exclusion columns (Izon Cat# SP5) followed by elution with PBS. 500 μl fractions were collected using an automatic fraction collector from Izon Science and Protein concentration was determined by NanoDrop A280. Only fractions that contained low protein concentrations were used for subsequent experiments. Purity, size, and concentration of all preparations of exosome fractions were analyzed using nano-tracking analysis (NTA) on a NanoSight LM10 (Malvern Panalytical). Particle counts and size was done with the NTA 3.1 software using a 50 μl/min flow rate and read at 25 °C on a 488 nM laser/filter. All NTA readings were performed using PBS which had been previously depleted of nanoparticle background by filtration through a 0.02 µM membrane.

### Exosome mass spectrometry

Exosome protein mass spectrometry was carried out as described by Pietrowska et al*.*^50^. Purified exosome was lysed by addition of 0.5 volumes lysis buffer (exoLB) consisting of 6% sodium dodecyl sulfate (SDS), 200 mM dithiothreitol (DTT), and 200 mM Tris–HCl pH7.6 at 95 °C for 5 min (2% SDS, 67 mM DTT, 67 mM Tris final). Addition of 0.25 volumes 400 mM iodoacetamide followed (100 mM final) and the sample then incubated for 45 min at room temperature in the dark. SP3 processing was then carried out similar to Moggrige et al.^51^. Two types of carboxylate-functionalized beads (Sera-Mag Speed Beads, GE Life Sciences, Cat# 45152105050350 and Cat# 65152105050350) were used (10 µl each/sample), twice rinsed in water using a magnetic rack for processing. Beads were added to exosome extracts, vortexed briefly, and acetonitrile added to a final concentration of 55% by volume, after which tubes were incubated at room temperature for 5 min with mixing at 1000 rpm in a ThermoMixer. Beads were then captured with a magnetic rack, supernatant discarded, and washed 3× with 200 µl 80% ethanol. After washes, tubes were removed from the magnetic rack and beads resuspended in 50 µl of 50 mM HEPES, pH 8 and 0.5 µg trypsin/LysC was added (Promega, Cat# V5071) and incubated overnight at 37 °C in a ThermoMixer with mixing at 1200 rpm. Beads were removed magnetically, the supernatant acidified with 5 µl 1% trifluoroacetic acid (TFA), and desalted using 200 µl TopTips, conditioned with 2 × 50 µl acetonitrile (ACN)/0.1% TFA, 2 × 50 µl water/0.1% TFA prior to sample loading. This was followed by 3 × 50 µl 0.1% formic acid (FA)/water washes and 2 × 75 µl 70% ACN/0.1% FA elution. The eluate was dried using a vacuum centrifuge (Centrivap), the residue dissolved in 10 µl 0.1% TFA, centrifuged 5 min 20,000×*g* and supernatant transferred to the sample plate.

Analysis of peptides was carried out on an Easy-nLC 1200/Orbitrap Fusion Lumos MS platform (Thermo Scientific control software version 3.1.2412.17). An in house prepared, 75 µm ID × 15 cm column (1.9 µm Dr Maisch) was equilibrated at 650 bar for a total volume of 8 μl and loading volume was 6 μl at 650 bar with 2 µl sample volumes. A gradient of mobile phase A (water and 0.1% formic acid) and B (80% acetonitrile with 0.1% formic acid) at 0.3 µl/min, 2–28%B from 2 to 92 min followed by 28–40% B, 92–102 min; 40–95% B, 102–108 min; 95%, 108–120 min; 50 °C was used for peptide elution.

A data dependent acquisition method with Orbitrap MS2 and 3 s cycle time was used. The Lumos was operated with a positive ion spray voltage of 2000, transfer tube temperature 325 °C, default charge state 2 with survey scans (MS1) acquired in the Orbitrap at 60 K resolution, m/z 375–1500, RF lens setting 30, AGC target 4e5, max injection time 50 ms in profile mode. MS2 parameters included precursor selection of 1.2 m/z, resolution 15 K, intensity threshold of 5e4, charge state filtering 2–5, dynamic exclusion 15 s with 10 ppm tolerances, HCD fragmentation at 33%, fixed first mass of 110 m/z, AGC target of 5e4, and a max injection time of 100 ms in centroid mode with parallelizable time turned off.

All data files were processed with Protein Discoverer 2.2.0.388. Spectrum files were recalibrated and features extracted with Minora. Searches were carried out with Sequest HT with SwissProt TaxID = 9606 (v2017-10-25) with precursor mass tolerance 10 ppm and fragment mass tolerance 0.01 Da with C carbamidomethyl as permitted fixed and M,P oxidation as permitted dynamic peptide modifications and acetyl *N*-terminal protein modification. Decoy database strict and relaxed FDR targets were 0.01 and 0.05 based on q value. Precursor quantification was intensity based with unique and razor peptides used, normalizing on total peptide amount with scaling on all average, and protein lists exported to Excel for any further analysis.

## Supplementary Information

Supplementary Figure S1.

Supplementary Figure S2.
