# Supplementary figures and images for: Internalization and trafficking of CSPG-bound recombinant VAR2CSA lectins in cancer cells
Source: Sci Rep. 2022 Feb 23;12:3075. doi: 10.1038/s41598-022-07025-6 (PMC8866492; doi:10.1038/s41598-022-07025-6)

Figure 1b

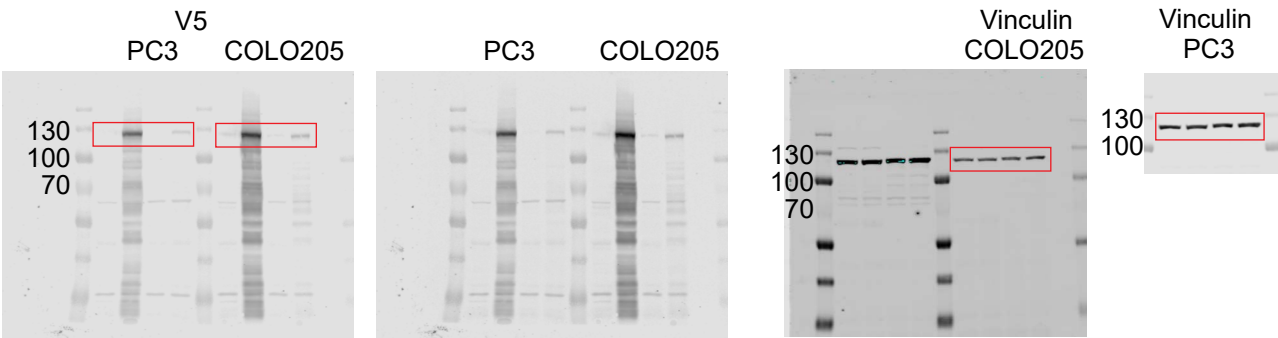

Figure 1f

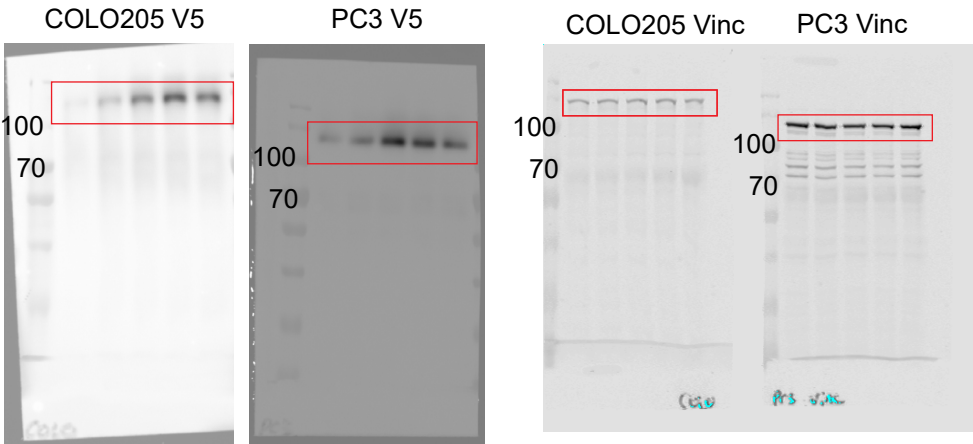

Figure 2c

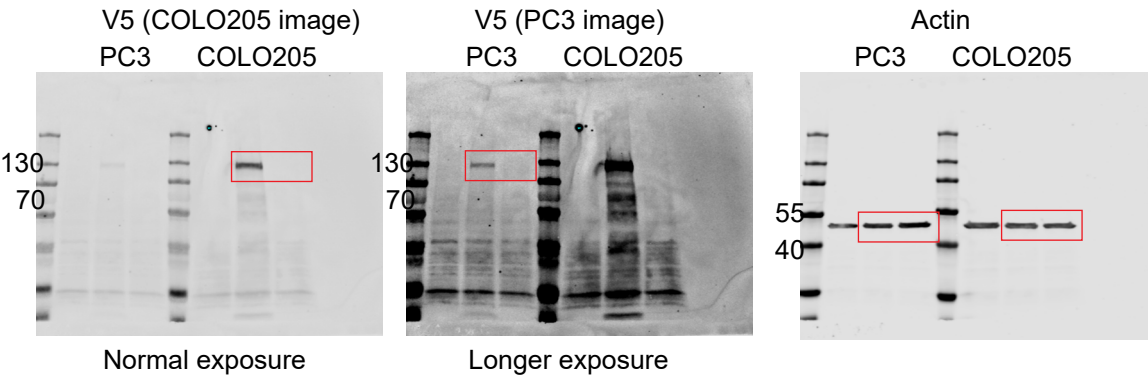

Figure 2e

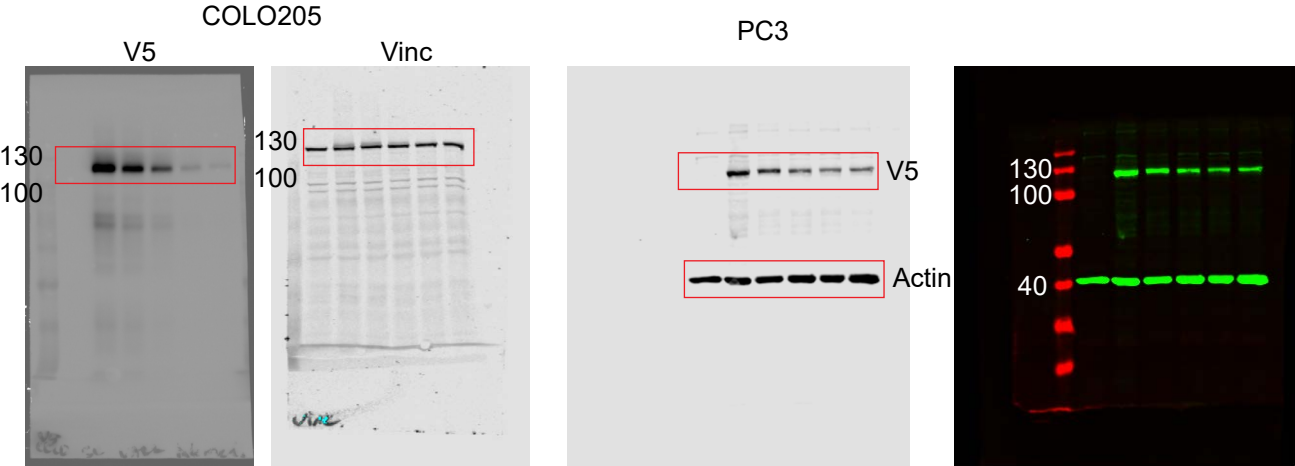

Figure 2i

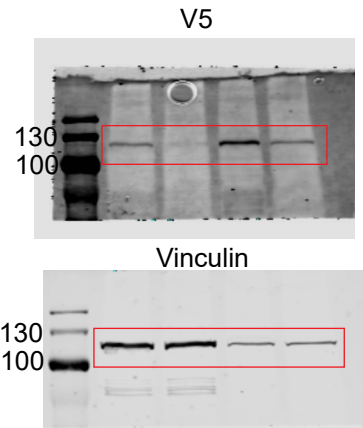

Figure 3a

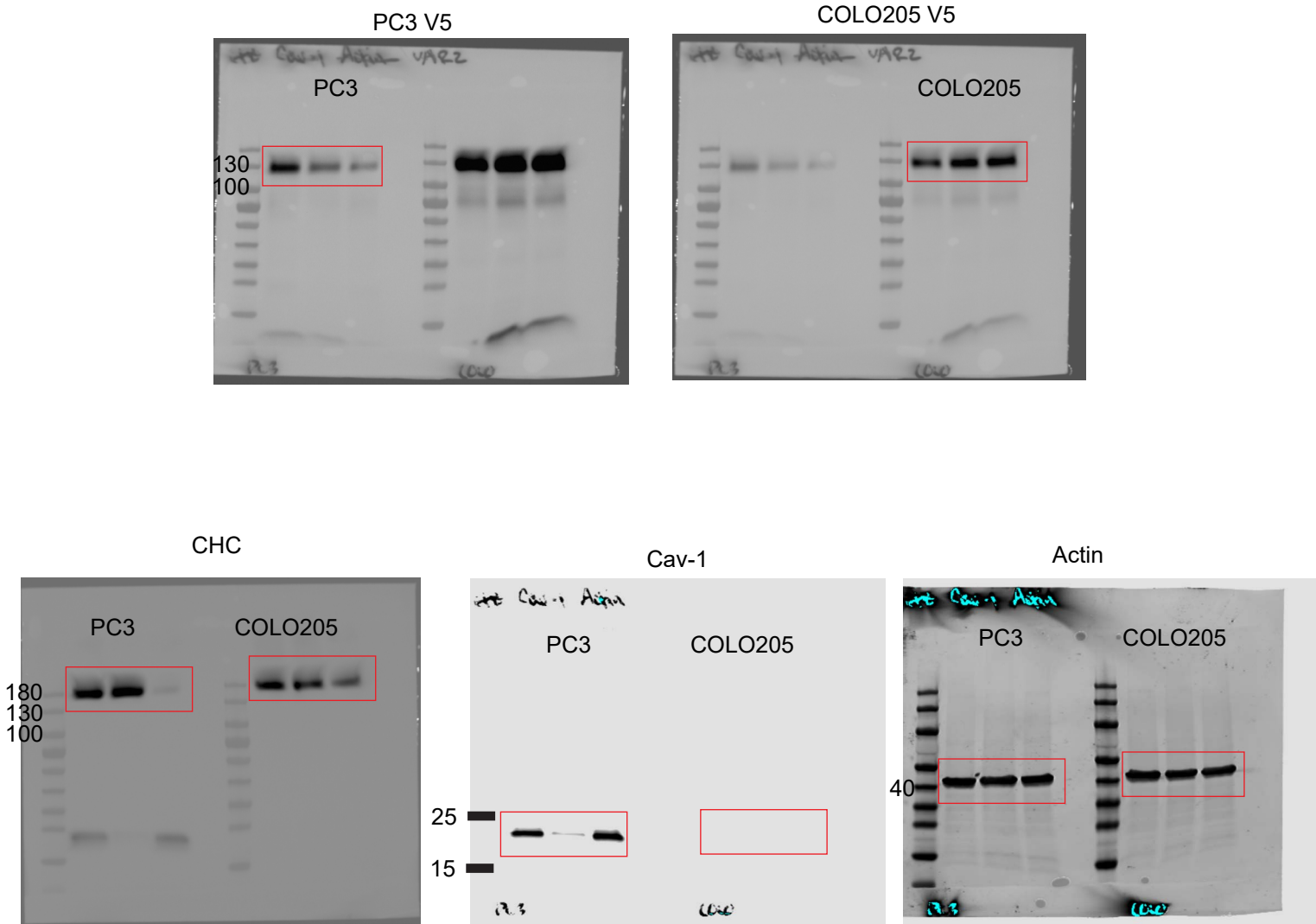

Figure 4c

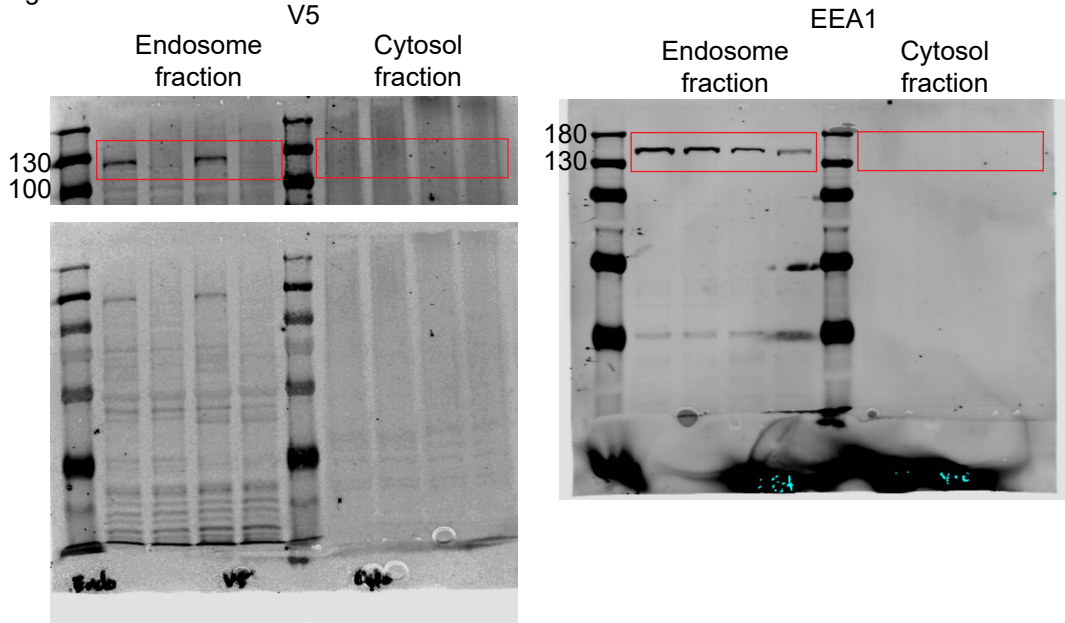

Figure 5a

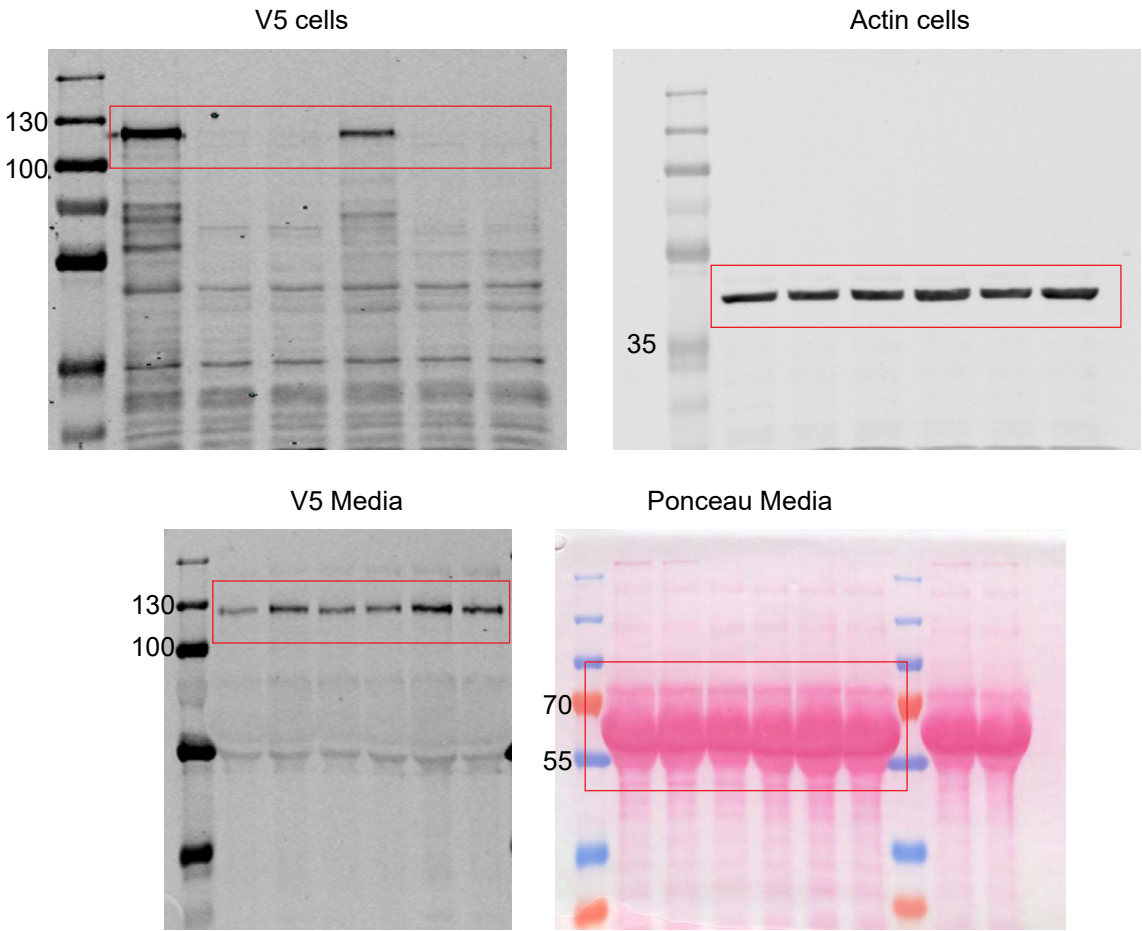

Figure 5e

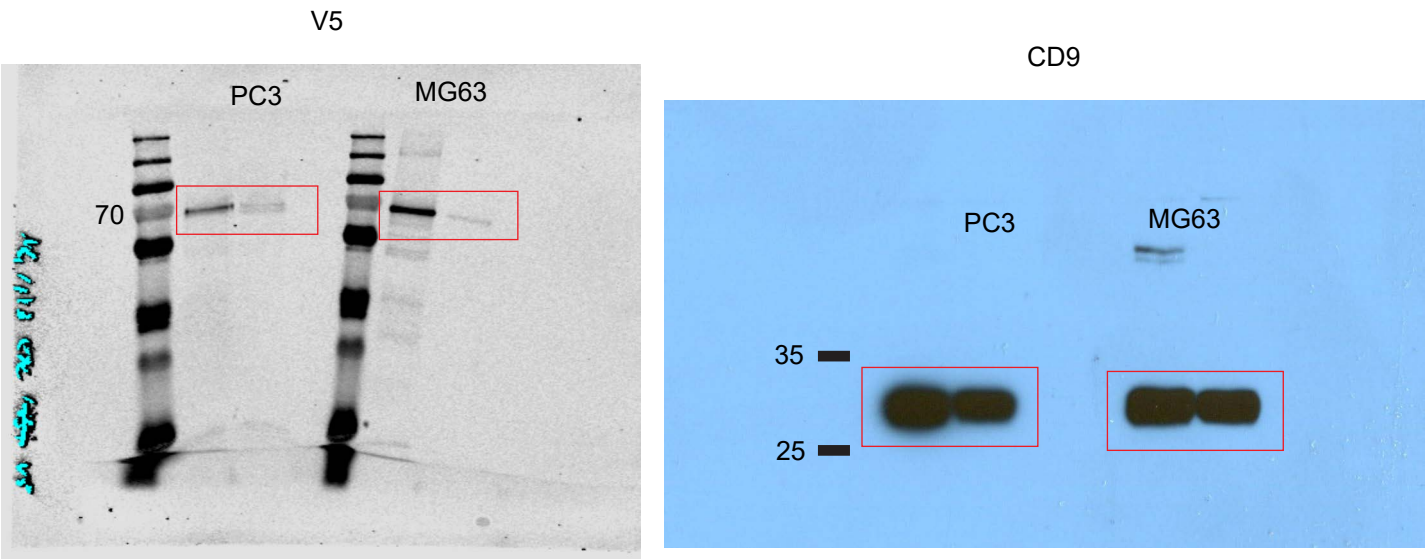

Supplement: Supplementary file 1 — Supplementary Figure S1. [file 41598_2022_7025_MOESM1_ESM.pdf]
