# Supplementary material for: Internalization and trafficking of CSPG-bound recombinant VAR2CSA lectins in cancer cells
Source: Sci Rep. 2022 Feb 23;12:3075. doi: 10.1038/s41598-022-07025-6 (PMC8866492; doi:10.1038/s41598-022-07025-6)
Supplement: Supplementary file 2 — Supplementary Figure S2. [file 41598_2022_7025_MOESM2_ESM.pdf]

Supplementary Figure S2

A

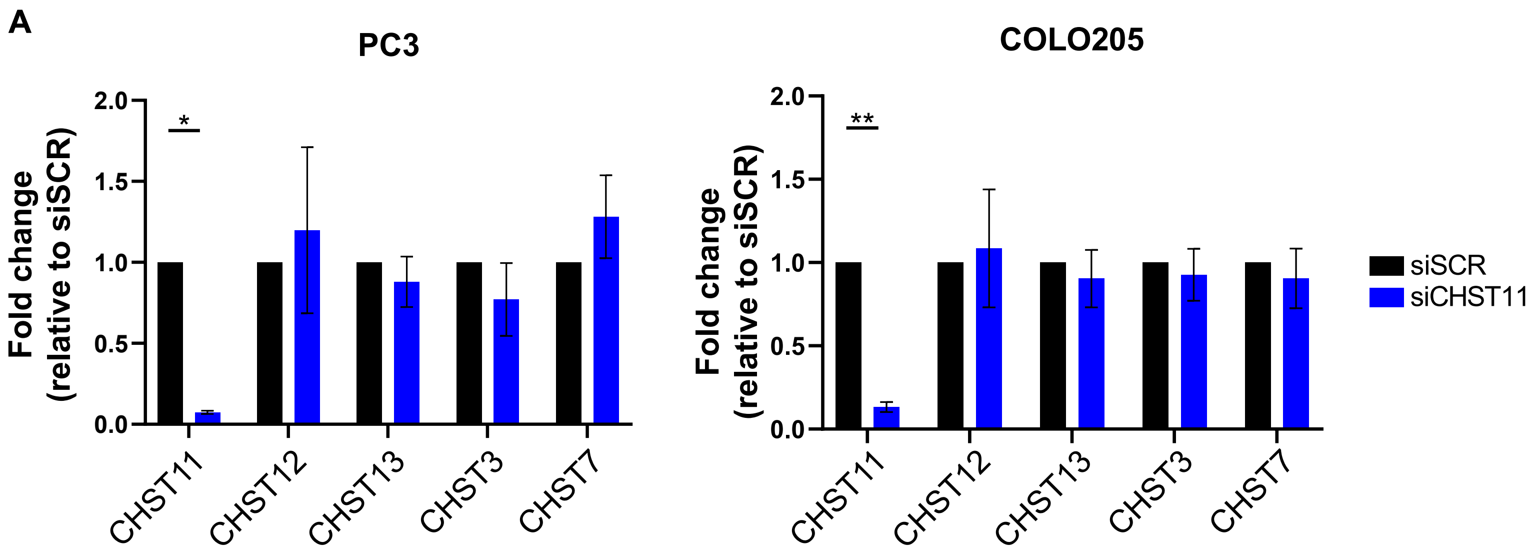

Figure S2a. Sulfotransferase expression after CHST11 knockdown. qPCR for sulfotransferases following siCHST11 in Fig 2g, 2h, and 2i. mRNA levels are were normalized to GapDH. Data represent mean±SEM of three independent biological replicates. * = p<0.05, ** = p<0.01

B

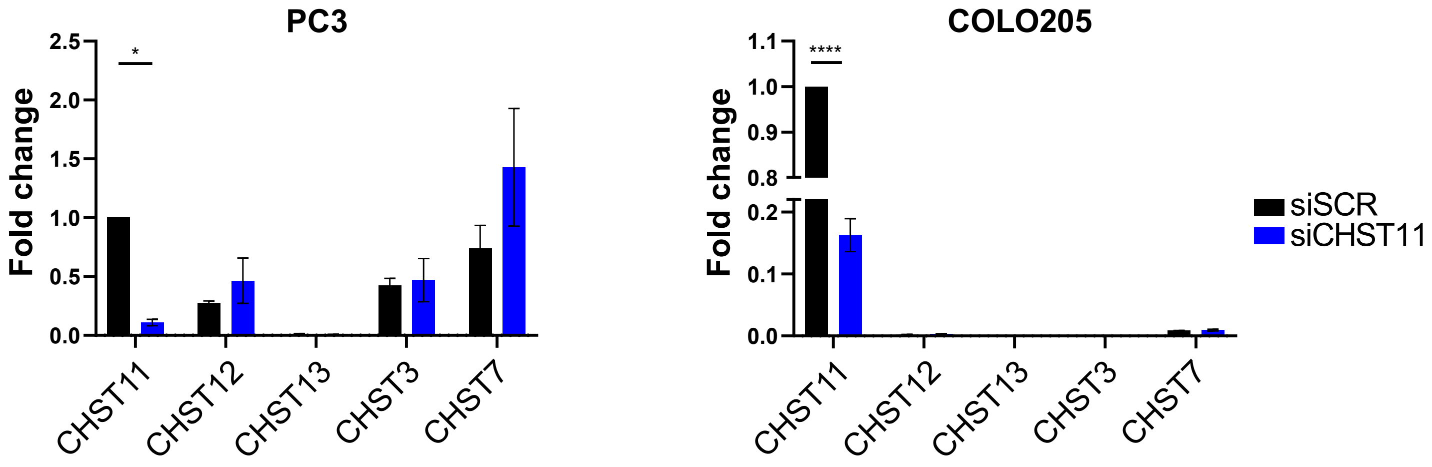

	siSCR			siCHST11		
	R1	R2	R3	R1	R2	R3
CHST11	1.000	1.000	1.000	0.055	0.142	0.129
CHST12	0.308	0.270	0.244	0.131	0.463	0.799
CHST13	0.022	0.002	0.001	0.012	0.004	0.002
CHST3	0.533	0.409	0.336	0.214	0.826	0.372
CHST7	0.482	0.608	1.124	0.432	1.840	2.013

	siSCR			siCHST11		
	R1	R2	R3	R1	R2	R3
CHST11	1.0000000	1.0000000	1.0000000	0.1546271	0.2124370	0.1215539
CHST12	0.0017464	0.0028186	0.0023466	0.0032802	0.0040183	0.0014632
CHST13	0.0009491	0.0002488	0.0000871	0.0008298	0.0002039	0.0001659
CHST3	0.0005376	0.0000527	0.0002358	0.0006832	0.0000653	0.0001336
CHST7	0.0089436	0.0075028	0.0087161	0.0120438	0.0086750	0.0074009

Figure S2b. Sulfotransferase expression following siCHST11 in PC3 and COLO205 cells. All genes were normalized to CHST11 of siSCR with respect to each cell line. Raw values of three biological replicates are provided in tables below each graph. Data represent mean±SEM of three independent biological replicates. * = p<0.05, **** = p<0.0001

C

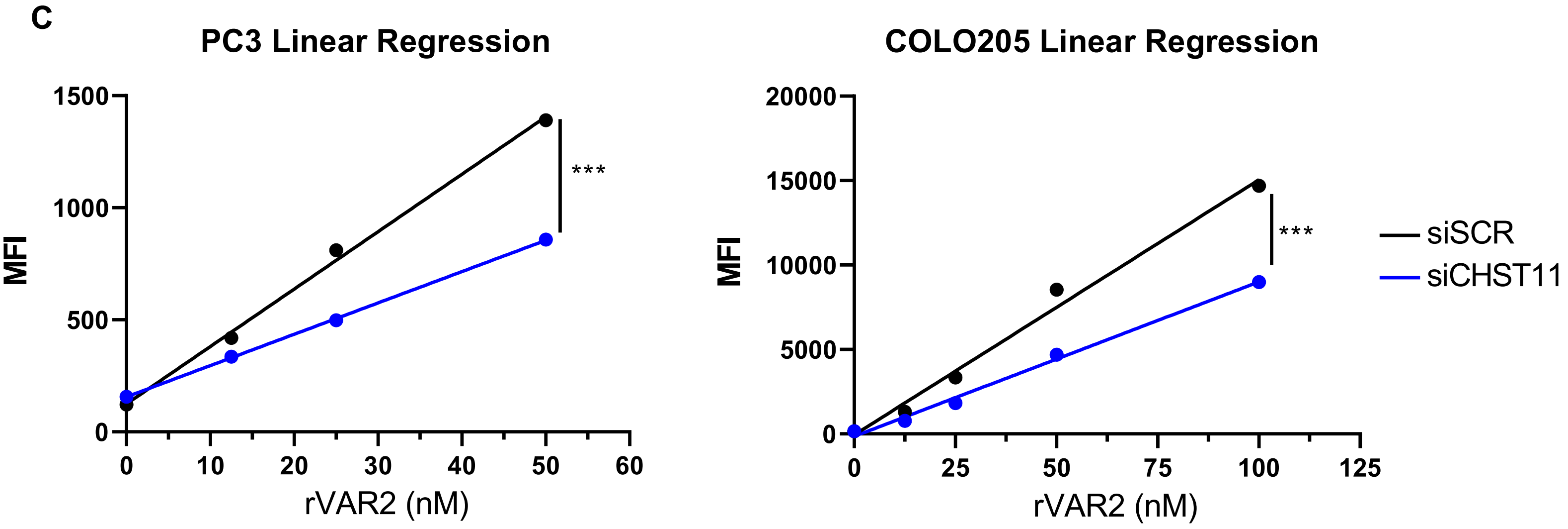

Figure S2c. Linear regression analysis of rVAR2 binding to PC3 and COLO205 cells after CHST11 RNAi of Fig. 2h. *** = p ≤ 0.001
